# Impact of diverse chemotherapeutic agents and external factors on activation of brown adipose tissue in a large patient collective

**DOI:** 10.1038/s41598-018-37924-6

**Published:** 2019-02-13

**Authors:** Cornelia Brendle, Norbert Stefan, Irina Stef, Sabine Ripkens, Martin Soekler, Christian la Fougère, Konstantin Nikolaou, Christina Pfannenberg

**Affiliations:** 1 Diagnostic and Interventional Radiology, Department of Radiology, Eberhard Karls University, Hoppe-Seyler-Straße 3, 72076 Tuebingen, Germany; 2 Diagnostic and Interventional Neuroradiology, Department of Radiology, Eberhard Karls University, Hoppe-Seyler-Straße 3, 72076 Tuebingen, Germany; 3 Endocrinology and Diabetology, Department of Internal Medicine, Eberhard Karls University, Otfried-Mueller-Straße 10, 72076 Tuebingen, Germany; 4 Oncology, Hematology, Clinical Immunology, Rheumatology and Pulmology, Department of Internal Medicine, Eberhard Karls University, Otfried-Mueller-Straße 10, 72076 Tuebingen, Germany; 5 Nuclear Medicine and Clinical Molecular Imaging, Department of Radiology, Eberhard Karls University, Otfried-Mueller-Straße 14, 72076 Tuebingen, Germany

## Abstract

Increased activity of brown adipose tissue (BAT) activity in adults is thought to prevent obesity. Therefore, regulators of BAT activity might serve as anti-obesity therapy in future, but are not investigated thoroughly up to now. In our study, we assessed retrospectively the association of BAT activity with several external factors and diverse chemotherapeutic and immunosuppressive agents in a collective of 702 patients. The patients underwent at least two clinically indicated PET/CT examinations in the course of different oncological and inflammatory diseases. BAT activity was identified according to predefined PET/CT criteria in all examinations. In multivariate analysis, the type of disease, the disease activity and the therapeutic regimen did not influence BAT activity. In contrast, sex and age were confirmed as independent factors for BAT activity. For the association of therapeutic agents with BAT activity, we examined 53 different disease-related agents, which were applied to patients without initial BAT activity between their PET/CT examinations. Out of these, cytarabine therapy was significantly associated with increased new onset of BAT activity. Cytarabine is a therapeutic agent for lymphoma patients. Further targeted studies might investigate the usefulness of Cytarabine serving as possible therapeutic approach against obesity via BAT regulation.

## Introduction

Brown adipose tissue (BAT) is found in humans and is predominantly located in the cervical and thoracic regions. Because of it’s high glucose uptake, active BAT can be identified by positron-emission-tomography (PET) using the glucose derivate 18F-Fluorodeoxyglucose (FDG). BAT contains uncoupling protein 1 in its mitochondria, which enables heat production by uncoupling oxidative phosphorylation from adenosine triphosphate (ATP) synthesis^1^. Newborns are capable of non-shivering thermogenesis by active BAT. BAT activity is also found in a minority of adults in thermo neutral conditions. It is considered a target to regulate body weight. This hypothesis is based on the observation that active BAT oxidizes large amounts of glucose and lipids, resulting in increased whole-body energy expenditure^2^. Accordingly, BAT activity is negatively associated with obesity and insulin resistance^3–5^.

Several factors and conditions have been found to correlate with BAT activity and some of them might potentially be useful for regulating BAT activity in a therapeutic setting. First of all, low outside temperature or cold exposition activate BAT via beta-noradrenergic receptors with subsequent activation of uncoupling protein 1^6–8^. Capsinoids and beta-adrenergic agonists like mirabegron increase BAT activity by the same pathway^9,10^. Other activators of uncoupling protein 1 are Irisin, thiazolidinediones and Imatinib, partially via PPARγ^11–13^. Glucocorticoids seem to have a controversial role for BAT activity. While BAT activity is elevated during short-term use, glucocorticoids have an inhibitory effect on BAT on long-term application^14,15^. Thyroid hormones show a positive correlation with BAT activity^16,17^. Isoflurane and beta-antagonists are known to have inhibitory effects on BAT activity^14^. Well-known patient inherent factors associated with BAT activity are age and sex. The prevalence of active BAT is negatively correlated with age and is higher in females than in males, possibly influenced by sex hormones^3,18^. There are also reports indicating that specific types of diseases influence BAT activity^19,20^. But, so far, not all changes of BAT activity can be explained by the known mechanisms.

The aim of our study was to identify possible unknown regulators of BAT activity. For this purpose, we assessed the association of BAT activity with several external factors including different chemotherapeutic and immunosuppressive agents in a large cohort of patients with diagnostic 18F-FDG-PET/computer tomography (CT) examinations.

## Materials and Methods

### Patients

We reviewed a total of 4852 clinically indicated 18F-FDG-PET/CT examinations in our institution between 08/2004 and 07/2009. For the present analysis, we included only data of patients with two ore more PET/CT examinations in order to enable the assessment of emerging BAT activity between two examinations. All patients were included irrespectively of their status of disease and possible prior treatment. We noted patient age, sex, height and weight and calculated the body mass index. The primary diseases of the patients were summarized into groups in order to ensure sufficient patient numbers for a statistical analysis. All patients gave their written informed consent for the scientific use of their data. The study was approved by the Ethics Committee of the Faculty of Medicine at the Eberhard Karls University Tuebingen. The study was performed in accordance with the Declaration of Helsinki.

### PET/CT acquisition and data analysis

All PET/CT examinations were performed with a state of the art PET/CT scanner (Hi-rez Biograph 16, Siemens Healthcare, Knoxville, USA) using standardized examination conditions and acquisition parameters under thermo neutral conditions, as explained before^21^. We loaded the PET/CT examinations to a viewing software (Syngo TrueD, Siemens Healthcare, Erlangen, Germany) and evaluated the fused datasets regarding BAT activity. Datasets were identified as displaying BAT activity, if a maximal FDG accumulation above the standard uptake value of 2.0 was found in symmetric regions paravertebral, nuchal, supra- or infraclavicular, and if the PET positive regions were projected on fat tissue in CT with Hounsfield units between −250 and −50^21^. The approach of BAT definition in this study represents a simple rough estimation of the BAT activity. Exact quantification of BAT activity and the assessment of dynamic tracer uptake might reflect the metabolic activity of BAT more precisely, but were not appropriate in this retrospective study with a large patient collective.

We noted the BAT activity status of the patients at the time point of the first PET/CT examination and at the follow-up examinations. In patients without initial BAT activity and new onset of BAT activity at a later examination, we noted details of the disease-related interim therapy (including specific chemotherapeutic or immunosuppressive agents) between the last examination without BAT activity and the first examination with BAT activity, in order to analyse the association of therapy with BAT activity. In patients without BAT activity at all examinations, we noted the therapy between the first and second PET/CT examination as control. For the disease activity status, we classified a disease as active, if any disease-related therapy was performed between the PET examinations; otherwise it was classified as inactive disease. The months of the dates of all PET/CT examinations were noted for the evaluation of possible seasonal changes of BAT activity.

### Statistical analysis

We used non-parametrical tests because the data was not normally distributed. We assessed the differences of BAT activity between sexes, months of examination dates, disease activity, prior therapy status, disease groups and therapy strategy as well as cytostatic agents using Chi square test in case of large sample sizes and using Fisher’s exact test in case of small sample sizes. Differences of BAT activity depending on age and body mass index at the second PET/CT examination as well as differences between the body mass indexes between the examinations and dependent on BAT activity were tested with Wilcoxon test. We set the significance level at p-value < 0.05. We performed Bonferroni correction for multiple testing. In order to separate confounder from independent factors associated with BAT activity, we performed multiple logistic regression analysis for all significant parameters in univariate analysis with exception of body mass index, because this is a dependent variable. Finally, we calculated the Odds ratio for independent factors.

## Results

### Patients

We included a total of 702 patients with repeated PET/CT examinations in the study (418 male, 284 female, mean ± standard deviation age 54 ± 16 years, body mass index 25.2 ± 4.6). The patients suffered from the following primary diseases: lymphomas (hodgkin lymphoma, non-hodgkin lymphoma, leukaemia, n = 144), gastrointestinal tumours (esophagus, stomach, intestine, n = 133), melanoma (n = 93), lung cancer (n = 73), thyroid cancer (n = 57), head and neck tumours (larynx, pharynx and sinuses, n = 39), gynaecological tumours (ovaries, uterus, n = 24), germ cell tumours (seminoma and non-seminomas of the testicles, n = 24), breast cancer (n = 22), cancer of unknown primary (n = 11), sarcoma (n = 10), tumours of the upper abdominal organs (pancreas, kidneys, gall bladder, n = 6), vasculitis (n = 38), inflammatory diseases (infectious, fever of unknown origin, n = 12), and others (cancers of prostate, penis, retroperitoneal fibrosis, fibromatosis, mesothelioma, thymus carcinoma, squamous cell carcinoma of the eye, n = 16). 440 patients underwent two PET/CT examinations, 153 three examinations, 67 four examinations, 23 five examinations, 10 six examinations, 4 seven, 1 eight, 2 nine and 2 ten examinations. The time interval between the examinations, which where included in the assessment, was 7.0 ± 7.3 months (mean ± standard deviation). 527 of the patients (57%) had undergone prior therapy before the PET/CT examinations; hereof received 263 patients (37%) chemotherapy, 140 (20%) radiation therapy, 337 (48%) surgery and 75 (11%) other therapies.

### Primary BAT activity

A total of 30 patients (4.3%) showed BAT activity at the first PET/CT examination. At this time point, females showed more often BAT activity than males (8.8% versus 1.7%, p < 0.0008) and younger patients more often than older patients (mean ± standard deviation 41 ± 15 years versus 55 ± 15 years, p < 0.0008). The percentage of patients with BAT activity did not differ between active and inactive disease (p = 1.00) and between patients with and without any prior therapy (p = 0.4–1.00). The highest proportions of patients with BAT activity were found at PET/CT examinations performed in November (8.9%) and February (7.5%) and the lowest proportions in April (1.7%) and May (1.9%), but there were no significant differences between the months. Patients with sarcoma showed more often BAT activity than patients with lung cancer, gastrointestinal tumours, head and neck tumours, melanoma, thyroid cancer and vasculitis, but had also a significantly younger age (mean age ± standard deviation 39 ± 18 years versus 61 ± 9, 60 ± 11, 55 ± 16, 57 ± 12, 55 ± 17 and 59 ± 15 years respectively). Patients with gynaecological tumours and breast cancer showed more often BAT activity than patients with head and neck tumours and vasculitis in the whole patient collective and in the female subgroup, with no significant differences regarding age. In multiple logistic regression analyses, sex and age remained as independent factors of BAT activity at the initial PET/CT examination (p < 0.0001, Odds ratio females/males = 4.92 [95% confidence interval 2.05–11.77] and p < 0.0001, Odds ratio = 0.02 [95% confidence interval 0.01–0.13], respectively). The affiliation to any disease group was not an independent parameter for BAT activity. For the following analyses, we excluded the patients with initial BAT activity.

### Secondary BAT activity and its association with therapy

BAT activity occurred in the course of the follow-up PET/CT examinations in 38 (5.7%) of 672 patients without initial BAT activity. In these patients, individuals with BAT activity were significantly younger than individuals without BAT activity (mean age ± standard deviation 38 ± 20 years versus 56 ± 14 years, p < 0.0008) and had a lower body mass index (mean ± standard deviation 22.3 ± 4.0 versus 25.5 ± 4.6, p < 0.0008), while BAT activity was independent from sex (p = 0.10) and disease activity (p = 0.68). The highest proportions of new onset of BAT activity were found at PET/CT examinations in the months October (13.0%) and February (8.2%), and the lowest proportions in the months March (0%) and May (1.5%), with a significant difference between March and October (p = 0.007). The body mass index of the patients did not differ significantly between the two assessed PET examinations (mean ± standard deviation 25.4 ± 4.8 versus 25.3 ± 4.7, p = 0.88) and the decrease of the body mass index between both PET examinations did not differ significantly in patients with and without BAT activity at the follow-up examination (mean ± standard deviation difference 0.06 ± 1.7 versus 0.05 ± 1.9, p = 0.96). The treatment strategies between the first and the follow-up PET/CT examinations consisted of chemotherapy (n = 282, hereof with BAT activity n = 19), radiation therapy (n = 114, with BAT activity n = 5), surgery (n = 139, with BAT activity n = 8) and/or other therapy, including immunotherapy or hormone therapy (n = 79, with BAT activity n = 7). Some patients received no therapy (n = 169) or combined therapies (n = 103). The incidence of BAT activity did not differ between patients with different therapeutic strategies (each p = 1.00). In chemotherapy, 53 different agents were used, usually as combined therapy in diverse combinations (see Table 1). The specific chemotherapeutic agents were unknown in 48 patients. Patients with administration of steroids (p < 0.0008), vincristine (p = 0.002), doxorubicin (p = 0.006), cytarabine (p < 0.0008) and vindesine (p = 0.04) showed a significantly higher incidence of BAT activity compared to patients without administration of these agents. Other chemotherapeutic or immunosuppressive agents were not associated with emerging BAT activity (see Table 1). In multiple regression analyses, age (Odds ratio = 0.02 [95% confidence interval 0.00–0.08], p < 0.0001), sex (Odds ratio female/male = 2.28 [95% confidence interval 1.10–4.71], p = 0.02) and cytarabine therapy (Odds ratio = 6.75 [95% confidence interval 2.20–20.65], p = 0.002) were independent parameters of BAT activity.

**Table 1:** Number of treated patients and association of chemotherapeutic and immunosuppressive agents with secondary BAT activity.

	Overall (n)	BAT activity (n)	p-value	Effect on BAT
**Alkylating agents**				
Cyclophosphamide	62	8	0.11	
Ifosfamide	28	2	1.00	
Dacarbazine	11	1	1.00	
Procarbazine	11	2	1.00	
Melphalan	9	1	1.00	
Carmustine	5	1	1.00	
Temozolomide	3	1	1.00	
Thiotepum	2	0	1.00	
Bendamustine	2	0	1.00	
**Platinum-based agents**				
Cisplatin	79	3	1.00	
Carboplatin	21	1	1.00	
Oxaliplatin	16	1	1.00	
**Anthracyclines**				
Doxorubicin	47	8	0.006*	activation
Epirubicin	9	0	1.00	
Daunorubicin	2	1	0.92	
Idarubicin	1	0	1.00	
Mitoxantrone	1	0	1.00	
**Peptid antibiotics**				
Bleomycin	15	2	1.00	
Mitomycin C	13	2	1.00	
Actinomycin D	1	0	1.00	
**Vinca alkaloids**				
Vincristine	50	9	0.002*	activation
Vinorelbine	14	0	1.00	
Vindesine	7	3	0.045*	activation
Vinblastine	6	1	1.00	
**Taxanes**				
Paclitaxel	29	1	1.00	
Docetaxel	5	0	1.00	
**Inhibitors of Topoisomerases**				
Etoposide	53	7	0.15	
Irinotecan	3	0	1.00	
Topotecan	1	0	1.00	
**Nucleotied analogs and precursor analogs**				
Fluoruracil	37	0	0.93	
Cytarabine	20	7	<0.0008*	activation
Methotrexate	18	3	0.67	
Azathioprine	6	0	1.00	
Fludarabine	5	0	1.00	
Gemcitabine	5	0	1.00	
Capecitabine	4	1	1.00	
Tioguanine	1	1	0.47	
Mercaptopurine	1	1	0.47	
Cladribine	1	0	1.00	
Clofarabine	1	1	0.47	
**Other**				
Steroids	64	12	<0.0008*	activation
Rituximab	42	4	1.00	
Other mAb	13	0	1.00	
Imatinib	13	0	1.00	
Motesanib	8	0	1.00	
Mycophenolate Mofetil	6	1	1.00	
Cyclosporin A	2	0	1.00	
Leflunomide	2	0	1.00	
Tacrolimus	2	0	1.00	
Everolimus	2	0	1.00	
Tamoxifen	2	0	1.00	
Anastrozole	1	0	1.00	
Sirolimus	1	1	0.47	

N, number of patients; *significant; mAb, monoclonal antibody.

### Secondary BAT activity within different disease groups

All patients with cancer of unknown primary or gynaecological tumours, germ cell tumours, tumours of the upper abdomen and inflammatory diseases remained in a status without BAT activity. Patients with lymphoma showed BAT activity significantly more often than patients with lung cancer and gastrointestinal tumours. BAT activity did not differ significantly between the other disease groups. The patient characteristics in the different disease groups are shown in Table 2. The affiliation to a certain disease group was no independent factor for new onset of BAT activity in multiple regression analysis.

**Table 2:** Characteristics of patients without initial BAT activity in the different disease groups.

	Overall (n)	BAT activity^a^ (n)	Mean age (years)^b^	Male/Fe-male (n)	Active disease (n)
Lung cancer	71	1	61 ± 9	53/18	58
Cancer of unknown primary	11	0	64 ± 11	4/7	7
Gastrointestinal tumours	128	4	60 ± 11	94/34	110
Gynaecological tumours	20	0	59 ± 8	0/20	14
Head and neck tumour	39	2	55 ± 16	26/13	24
Germ cell tumour	23	0	42 ± 9	23/0	15
Lymphoma	135	17	45 ± 19	86/49	105
Breast cancer	18	1	54 ± 12	0/18	11
Melanoma	92	5	57 ± 12	62/30	61
Cancer of the upper abdomen	6	0	72 ± 8	2/4	3
Sarcoma	8	1	38 ± 19	5/3	7
Thyroid cancer	55	4	55 ± 17	27/28	36
Inflammatory disease	12	0	56 ± 14	10/2	7
Vasculitis	38	2	59 ± 15	10/28	35
Other	16	1	54 ± 16	8/8	8
Total	672	38	55 ± 15	411/261	501

N, number of patients;

^a^new onset of BAT activity during follow-up examinations.

^b^mean age in years ± standard deviation.

Sex, disease activity and the therapy regimen were not associated with BAT activity within any of the disease groups (p = 0.11–1.00). While different age, body mass index and specific therapeutic agents did not associate with BAT activity within the most disease groups, they were correlated with BAT activity in lymphoma patients (n = 135). In detail, lymphoma patients with younger age (p < 0.0007) and lower body mass index (p = 0.02) showed higher BAT activity. Furthermore, the administration of steroids (p = 0.04) and cytarabine (p = 0.008) resulted in significantly increased incidence of BAT activity in lymphoma patients. Age and cytarabine therapy remained as independent factors for BAT activity in patients in the lymphoma group (p = 0.0001, Odds ratio = 0.01 [95% confidence interval 0.00–0.18] and p = 0.008, Odds ratio = 6.32 [95% confidence interval 1.65–23.45], respectively). Lymphoma patients were the only disease group receiving cytarabine therapy (20 of 135 patients). The mean age between lymphoma patients receiving cytarabine versus not receiving cytarabine did not differ significantly (mean age ± standard deviation 39 ± 17 years and 46 ± 19 years respectively, p = 0.1); the percentage of females was comparable in both groups (35% and 38% respectively, p = 0.7). Cytarabine was administered in combination with other chemotherapeutics as part of diverse therapy protocols (e.g. BEAM protocol, DHAP protocol, GMALL B-ALL/NHL-2002 study protocol, ALL-BFM 2000 protocol and others). The association of BAT activity with specific lymphomas and leukaemias is shown in Table 3.

**Table 3:** Incidence of secondary BAT activity in correlation to therapy in patients with lymphoma and leukaemia.

Disease	Cytarabine administration (n)		Other therapies (n)		No therapy^a^ (n)	
	Overall	BAT activity	Overall	BAT activity	Overall	BAT activity
NHL	9	1	25	1	25	—
Hodgkin lymphoma	3	2	28	6	12	1
ALL	3	3	—	—	1	—
AML	1	—	1	—	—	—
CLL	1	—	1	1	1	—
Unspecified lymphoma	3	1	10	1	7	—
Other	—	—	—	—	4	—
Total	20	7	65	9	50	1

N, number of patients; NHL, non-Hodgkin lymphoma; ALL, acute lymphatic leukaemia; AML, acute myelotic leukaemia; CLL, chronic lymphatic leukaemia.

^a^Corresponds to the term inactive disease in the manuscript.

## Discussion

BAT is potentially a protective factor against obesity, and the identification of agents influencing BAT activity might enable the development of new therapies against obesity. We assessed the association of BAT activity with different primary diseases as well as different chemotherapeutic and immunosuppressive therapies in a large retrospective patient cohort. The advantage of our approach is that it enables screening a relatively large group of patients for BAT activators under thermoneutral, and therefore, under “real-world” conditions.

Out of 53 different chemotherapeutic and immunosuppressive agents in our study, the administration of the cytostatic agent cytarabine was significantly associated with increased BAT activity. This is a novel finding. Patients receiving cytarabine showed new onset of BAT activity in 35% of cases, compared to 4.8% of the remaining patients. Cytarabine (synonyms ARA-C, cytosine arabinoside) is an antimetabolite in DNA synthesis and inhibits the function of the DNA polymerase^22–24^. It has cytotoxic, antiviral and immunosuppressive effects^22,23,25,26^. Cytarabine is used for the treatment of a broad spectrum of lymphomas and leukaemias, including acute myelotic leukaemia, coat cell lymphoma (B-NHL) and other recurrent aggressive lymphomas, as well as, acute lymphatic leukaemia and Burkitt lymphoma (B-NHL)^22^. In our study, cytarabine was identified as an independent variable for emerging BAT activity. Except for age, all other studied parameters, namely sex, disease activity and therapy strategy, were not associated with BAT activity in lymphoma patients.

Gilsanz *et al*. postulated that active lymphoma may suppress BAT activity. In their patient cohort, the prevalence of active BAT was lower at initial diagnosis of lymphoma than in the follow-up period with inactive disease^19^. However, it can be assumed that the patients received some kind of chemotherapy between both time points. The higher BAT activity at the second examination might at least be partially caused by therapy in synopsis with our findings. Conversely, it is less probable that active lymphoma suppresses BAT activity based on the findings of our study, because BAT activity did not differ between lymphoma patients and other disease groups. Within our lymphoma group, especially patients with Hodgkin lymphoma and acute lymphatic leukaemia had a high percentage of BAT activity under cytarabine administration; however the patient numbers of the specific diseases were too small for a statistical analysis. Recent investigations on the mechanism of cytarabine effect have shown, that cytarabine stimulates the AMP-activated protein kinase (AMPK) in specific leukemic cell lines leading to autophagy and cell cycle arrest, while it has no effect on other cell lines^27–30^. Interestingly, AMPK also stimulates the formation and activity of BAT and is important for the maintenance of the cell function in brown adipocytes^31–33^. Cytarabine might, therefore, be a more or less specific activator of BAT via AMPK. Thus, future studies might investigate the efficacy and specificity of AMPK signalling to activate BAT in humans.

Other agents associated with increased new onset of BAT activity in univariate analyses were steroids, doxorubicin and the vinca alkaloids vincristine and vindesine. However, none of these agents was identified as independent parameter of BAT activity in the multivariate analysis. Therefore, considerations on the impact of these agents on BAT activity can be only hypothetical based on our results. Glucocorticoids are thought to influence BAT activity in humans in a complex way via regulation of uncoupling protein 1 and AMPK activity^34–36^. Short-term administration of glucocorticoids acutely increases the proliferation and function of BAT, while long-term application suppresses BAT activity^14,37–39^. BAT activity tended to be higher after administration of glucocorticoids in our study. However, we did not assess the duration of glucocorticoid therapy and therefore cannot add evidence to this topic. The cytostatic doxorubicin tended to increase BAT activity in our study and has been reported being an activator of BAT thermogenesis by activation of p53 in an animal model^40^. Doxorubicin activity is inversely associated with AMPK activity^41,42^. Doxorubicin and its connection with the AMPK signal pathway and BAT activity might be an interesting topic to be examined in future studies. Concerning vincristine and vindesine, there are no reports about vinca alkaloids and BAT activity beyond the findings of our study. However, mutual influence of vincristine and AMPK activity has been reported^42–44^. Therefore, these agents and their association with BAT activity might be investigated in future studies.

Concerning the effect of different therapeutic strategies on BAT activity, Steinberg *et al*. depicted chemotherapy as a possible inhibitor for BAT in a large retrospective analysis with different diseases^7^. Contrarily, our study did not reveal any effect of different therapy regimens on BAT activity. However, a comparison between the studies is difficult, because Steinberg *et al*. did not specify the diseases and therapeutic agents of their patient collective in detail and therefore they might differ from our study.

Regarding the impact of different primary diseases on BAT activity, we found a high prevalence of BAT activity in sarcoma patients, which, however, may be explained by the young age of these patients. Previous studies reported that patients with breast cancer had a higher prevalence of active BAT^7,45,46^. This is at least partially reflected in our results. We found that patients with breast cancer and gynaecological tumors had a relatively high prevalence of BAT activity. Though, the affiliation to any primary disease was not an independent factor for BAT activity in multivariate analysis in our study, therefore we cannot confirm an effect of any disease on BAT activity. The time points of the PET/CT examinations were variable in the course of the diseases based on disease-related clinical indication, and time points of BAT activity might have been missed, Therefore, the effect of specific diseases on BAT activity cannot be thoroughly analysed in a study like ours. A negative effect of chemotherapy in breast cancer patients on BAT activity has been reported, but was not confirmed in the small number of breast cancer patients in our study^7,47^.

Previous studies reported associations between BAT activity and tumor-induced or therapy-related cachexia^48^. Based on our data, we did not find a higher weight loss in patients with new onset of BAT activity, however there was no relevant average weight-loss in our patient collective. An association of BAT activity with outside temperature and concomitantly season is well documented^3,6–8,49^. Our data showed no significant differences of BAT activity between examinations performed at summer and winter months. This is possibly due to air-conditioning in our waiting and examinations rooms with standardized temperatures balancing different outside temperatures.

Our study had several limitations. First, we did not investigate the patients after cold exposition as recommended by BARCIST^50^. This was due to the retrospective study design with a large patient collective focused on the clinical reality and not on an experimental setting. Also, cold exposure might not be suitable in the hypothetic case of considering BAT regulation as therapeutic approach against obesity. Second, the assignment of single diseases to the different disease groups was somewhat arbitrary. Furthermore, the PET examinations were performed at different stages of the diseases and at different treatment statuses. All of this could have introduced bias in the statistical analysis. There are several co-factors discussed as potential parameters connected with BAT activity and not all could be considered in this study. Inhibitory factors of BAT activity might not be revealed by our study due to the retrospective design and the overall low percentage of BAT activity in the patient collective. Tracer uptake in PET is merely an indirect and rough assessment of BAT activity Furthermore, an exact quantification of BAT activity in PET was not performed in this study, but could reveal more details about the extent of BAT activity regulation by certain factors.

In conclusion, disease activity, the type of oncologic or immunological disease and the therapy strategy did not influence BAT activity. However, out of a large number of investigated therapeutic agents, cytarabine turned out to be an independent factor associated with increased BAT activity in this screening study. Its interconnections with the AMPK pathway and possible usefulness for anti-obesity therapy by enhancing BAT activity might be a topic of further research. Further studies are needed as well for steroids, vinca alkaloids and doxorubicin and their possible connection with BAT activity.

## Data Availability

The datasets generated during the current study are available from the corresponding author on reasonable request.
